# Modulation of retinal capillary endothelial cells by Müller glial cell-derived factors

**Published:** 2009-02-27

**Authors:** Hayato Abukawa, Masatoshi Tomi, Jumpei Kiyokawa, Satoko Hori, Tetsu Kondo, Tetsuya Terasaki, Ken-ichi Hosoya

**Affiliations:** 1 Department of Pharmaceutics, Graduate School of Medicine and Pharmaceutical Sciences, University of Toyama, Toyama, Japan; 2 Department of Molecular Biopharmacy and Genetics, Graduate School of Pharmaceutical Sciences, Tohoku University, Sendai, Japan

## Abstract

**Purpose:**

The inner blood-retinal barrier (BRB) is a gliovascular unit in which macroglial cells surround capillary endothelial cells and regulate retinal capillaries by paracrine interactions. The purpose of the present study was to identify genes of retinal capillary endothelial cells whose expression is modulated by Müller glial cell-derived factors.

**Methods:**

Conditionally immortalized rat retinal capillary endothelial (TR-iBRB2) and Müller (TR-MUL5) cell lines were chosen as an in vitro model. TR-iBRB2 cells were incubated with conditioned medium of TR-MUL5 (MUL-CM) for 24 h and subjected to microarray and quantitative real-time PCR analysis.

**Results:**

TR-MUL5 cell-derived factors increased alkaline phosphatase activity in TR-iBRB2 cells, indicating that paracrine interactions occurred between TR-iBRB2 and TR-MUL5 cells. Microarray analysis demonstrated that MUL-CM treatment leads to a modulation of several genes including an induction of plasminogen activator inhibitor 1 (*PAI-1*) and a suppression of an inhibitor of DNA binding 2 (*Id2*) in TR-iBRB2 cells. Treatment with TGF-β1, which is incorporated in MUL-CM, also resulted in an induction of *PAI-1* and a suppression of *Id2* in TR-iBRB2 cells.

**Conclusions:**

In vitro inner BRB model study revealed that Müller glial cell-derived factors modulate endothelial cell functions including the induction of anti-angiogenic *PAI-1* and the suppression of pro-angiogenic *Id2*. Therefore, Müller cells appear to be one of the modulators of retinal angiogenesis.

## Introduction

The inner blood-retinal barrier (inner BRB) is a selectively permeable interface between the circulating blood and neural retina. It is essential for the maintenance of neural functions, since dysfunction of the inner BRB is closely involved in many retinal disorders including diabetic retinopathy [1,2]. The inner BRB is formed by retinal capillary endothelial cells that exhibit several distinctive characteristics, such as tight junctions and lack of fenestrations to restrict nonspecific transport. Transporters at the inner BRB play essential roles in supplying nutrients and are also responsible for the efflux of neurotransmitters and their metabolites.

The inner BRB has been proposed to be a gliovascular unit, in which macroglial cells surround retinal pericytes and capillary endothelial cells and regulate retinal capillaries by paracrine interactions [3,4]. The vascularized retina of primates and rodents contains two types of macroglial cells: Müller cells and astrocytes. Müller cells are distributed radially across all retinal layers, whereas astrocytes are found only on the vitreal side of the retina, within the nerve fiber and ganglion cell layers. An in vivo study demonstrated that implantation of cultured Müller cells into the anterior chamber of the rat eye induces vessels associated with implanted Müller cells to become impermeable to albumin and horseradish peroxidase [5]. In vitro studies have shown that the permeability of bovine retinal vascular endothelial cells is reduced by conditioned medium from Müller cells [6,7] but is increased by matrix metalloproteinase 9 (MMP9), the release of which from bovine retinal vascular endothelial cells is stimulated by direct contact with astrocyte and Müller cells [8]. However, the physiologic role and mechanism of the gliovascular unit at the inner BRB is only beginning to be understood.

The purpose of the present study was to identify differently expressed genes of retinal capillary endothelial cells in response to Müller cell-derived factors by performing a microarray analysis, which allows the detection of over 15,000 transcripts. Conditionally immortalized rat retinal capillary endothelial (TR-iBRB2) and Müller (TR-MUL5) cell lines were chosen as an in vitro model, since these cell lines maintain certain in vivo functions and are of the same maturational stage, strain, and genetic background [9,10]. Using these culture cell lines, it is possible to overcome problems posed by contamination by other types of cells, species differences, and the limited size of the organs.

## Methods

### Cell culture

TR-iBRB2 cells are a conditionally immortalized rat retinal capillary endothelial cell line and TR-MUL5 cells are a conditionally immortalized rat retinal Müller cell line. Both cell lines were established from transgenic rats harboring a temperature-sensitive SV 40 large T-antigen gene [9,10]. TR-iBRB2 cells possess endothelial markers and facilitative glucose transporter 1, P-glycoprotein, creatine transporter, and L-type amino acid transporter 1 that are expressed at the inner BRB in vivo [10-12]. TR-MUL5 cells possess typical Müller cell markers, such as S-100, glutamine synthetase, and excitatory amino acid transporter 1 [9,13,14]. These cell lines were cultured in Dulbecco’s modified Eagle’s medium (DMEM; Nissui Pharmaceuticals, Tokyo, Japan) supplemented with 10% fetal bovine serum (FBS; Moregate, Bulimbra, Australia) at 33 °C in a humidified atmosphere of 5% CO_2_/air. The permissive temperature for these cell lines to be cultured was 33 °C, due to the presence of temperature-sensitive SV40 large T-antigen. TR-iBRB2 cells were seeded onto a rat tail collagen type I-coated tissue culture dish (BD Biosciences, Bedford, MA).

### Interaction with TR-MUL5 cells and TGF-β1

**Figure 1:**
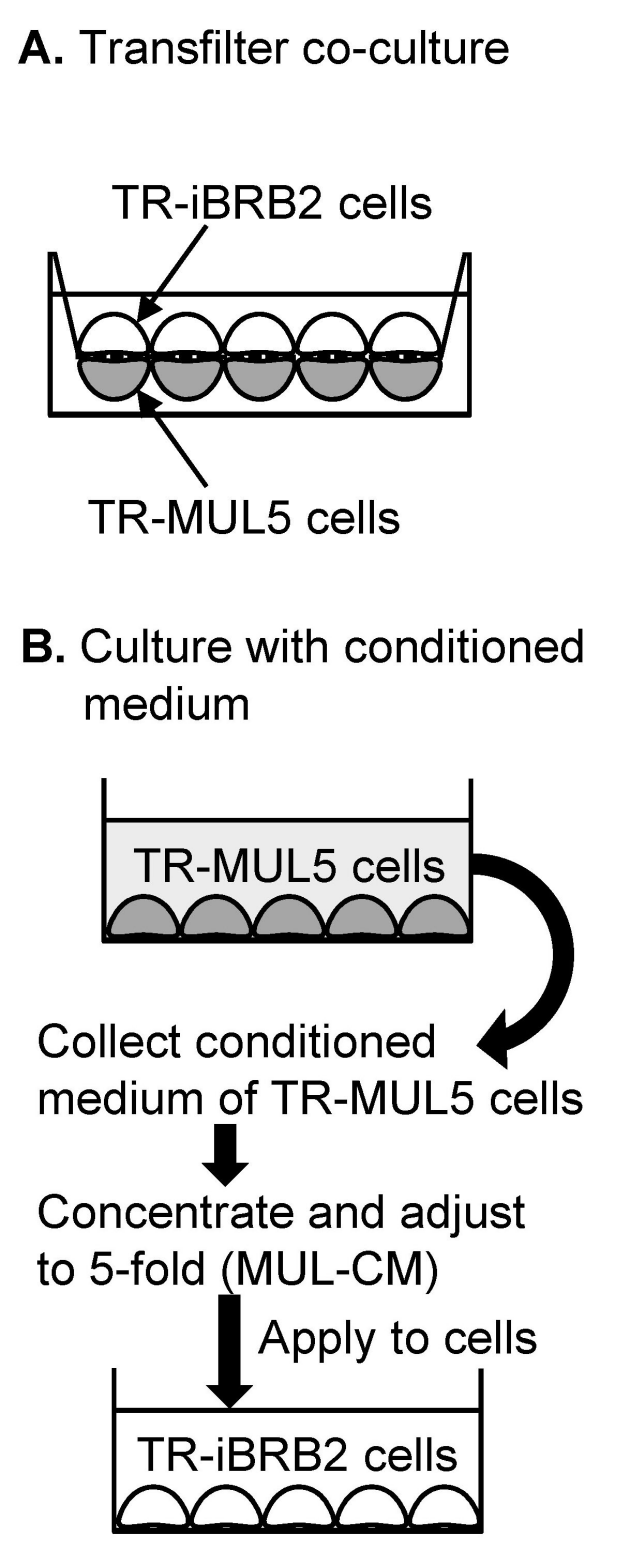
In vitro cell culture models. These models were used for analyzing paracrine interactions between retinal capillary endothelial and Müller cells. **A:** In the transfilter co-culture, TR-MUL5 cells were situated on the basolateral aspect of TR-iBRB2 cells monolayer as might occur in vivo. **B:** The fivefold concentrated conditioned medium of TR-MUL5 cells (MUL-CM) was applied to TR-iBRB2 cells.

In the transfilter co-culture, TR-MUL5 cells were seeded on the backside membrane of a rat tail collagen type I-coated cell culture insert (pore side: 3.0 μm, BD Biosciences) (Figure 1A). After a 24 h culture, the insert was transferred to a six well tissue culture plate. TR-iBRB2 cells were seeded on the upper side of the insert and were co-cultured for 6 days.

Conditioned medium of TR-MUL5 cells was prepared by incubating 4 ml of serum-free DMEM with TR-MUL5 cells for 24 h onto a 100 mm tissue culture dish (Figure 1B) [15,16]. The conditioned medium was passed through a 0.22 μm filter, concentrated up to 20 fold in a Centriprep or a Microcon (3 kDa cut-off; Millipore, Billerica, MA), and then stored at −20 °C until required. The conditioned medium was adjusted to fivefold concentrated conditioned medium of TR-MUL5 cells (MUL-CM) by diluting the 20 fold concentrated conditioned medium with serum-free DMEM. TR-iBRB2 cells were then treated with MUL-CM or 2 ng/ml of recombinant human transforming growth factor β1 (TGF-β1; R&D Systems, Minneapolis, MN), which shows 111/112 amino acid sequence identity with rat TGF-β1, for 24 h. It is demonstrated that 2 ng/ml human TGF-β increased MMP9 activity and the permeability of primary cultures of bovine retinal endothelial cells. Therefore, we chose the 2 ng/ml as a concentration of TGF-β1.

### Alkaline phosphatase activity

The alkaline phosphatase activity in TR-iBRB2 cells was measured using an alkaline phosphatase test kit (Wako Pure Chemicals, Osaka, Japan) according to the manufacturer’s protocol. Briefly, TR-iBRB2 cells were dissolved in phosphate buffered saline (PBS; 137 mM NaCl, 8.10 mM Na_2_HPO_4_, 2.68 mM KCl, and 1.47 mM KH_2_PO_4_) containing 0.5% Triton-X. Cell lysate was mixed with *p*-nitrophenyl phosphate in reaction buffer (pH 9.8) and was incubated at 37 °C for 15 min. *p*-Nitrophenyl phosphate is hydrolyzed into *p*-nitrophenol in the presence of alkaline phosphatase. Subsequently, the reaction was terminated by adding NaOH and the absorbance was measured at 405 nm. Protein concentrations were determined using a DC protein assay kit (Bio-Rad, Hercules, CA).

### Microarray

A GeneChip Rat Expression Array 230A (Affymetrix, Santa Clara, CA), which consists of 15,866 probe sets, was used in this experiment. Total cellular RNA was extracted from TR-iBRB2 cells using an RNeasy kit (Qiagen, Hilden, Germany) according to the manufacturer’s protocol. Poly(A)^+^ RNA was purified from total cellular RNA using Oligotex-dT30 super (Takara, Shiga, Japan) and cleaned by phenol/chloroform extraction. A T7-(dT)24 oligomer, superscript reverse transcriptase II, and DNA Polymerase I (Invitrogen, Carlsbad, CA) were used for first-strand and second-strand cDNA synthesis using Poly(A)^+^ RNA as templates. Double-stranded cDNA was cleaned with Phase Lock Gels-Phenol/Chloroform extraction and ethanol precipitation. Biotin-labeled antisense cRNA was produced by an in vitro transcription reaction (Enzo BioArray High Yield RNA Transcript Labeling Kit; Affymetrix) and incubated with fragmentation buffer. Fragmented biotin-labeled antisense cRNA was hybridized to the GeneChip in a GeneChip hybridization oven (Affymetrix). The hybridized GeneChip was washed and stained using a GeneChip fluidics station 400 (Affymetrix) and was then scanned by a GeneChip scanner (Affymetrix). The definitions of presence and absence of gene expression were defined by the Affymetrix GCOS 1.2 statistical algorithm. Gene expression analyses, including global normalization and scaling, were performed using the Affymetrix GCOS 1.2, Array Assist 3.01 software.

### Immunoblot analysis

Protein concentrations of MUL-CM were determined using a DC protein assay kit. The MUL-CM was mixed with sample buffer composed of 2% sodium dodecyl sulfate (SDS), 50 mM Tris-HCl (pH 6.8), 10% glycerol, 6% 2-mercaptoethanol, and 0.01% bromophenol blue. The mixture was heated for 10 min at 95 °C. Next, 100 μg protein of MUL-CM was electrophoresed on an SDS-polyacrylamide gel and, subsequently, electrotransferred to a polyvinylidene difluoride membrane. Following incubation with blocking agent solution (Block Ace; Dainihon Pharmaceutical, Osaka, Japan), the membranes were incubated with 1:300 mouse monoclonal anti-TGF-β1 antibody (R&D Systems) for 16 h at 4 °C. The membranes were subsequently incubated with horseradish peroxidase conjugated anti-mouse IgG. The bands were visualized using an enhanced chemiluminescence kit (GE Healthcare, Piscataway, NJ).

### Quantitative real-time PCR

Quantitative real-time polymerase chain reaction (PCR) was performed using an ABI PRISM 7700 sequence detector system (Applied Biosystems, Foster City, CA) with 2×5 ml SYBR Green PCR Master Mix (Applied Biosystems) according to the manufacturer’s protocol. Single-strand cDNA was made from total cellular RNA by reverse transcription (RT) using oligo dT primer. The control lacking the RT enzyme was assayed in parallel to monitor any possible genomic contamination. To quantify the amount of specific mRNA in the samples, we generated a standard curve for each run using the plasmid (pGEM-T Easy Vector; Promega, Madison, WI) containing the gene of interest. This enabled standardization of the initial mRNA content of cells relative to the amount of *β-actin*. The PCR was performed with plasminogen activator inhibitor 1 (*PAI-1*), inhibitor of DNA binding 2 (*Id2*), or *β-actin* specific primers (Table 1) through 40 cycles of 94 °C for 30 s, 60 °C for 30 s, and 72 °C for 1 min. The PCR products were separated by electrophoresis on an agarose gel and visualized under ultraviolet light to confirm the specificity of the primers for the target gene.

**Table 1:** Oligonucleotide primers used for PCR amplification of cDNAs.

**Target mRNA**	**Accession number**	**Primers (5′-3′)**	**Product size**
*PAI-1*	NM_012620	F: GAGGATGAAAGAAACAGCCAGCT	145 bp
		R: CCCGCTATGAAATTAGATTCACGT	
*Id2*	NM_013060	F: ATGAAAGCCTTCAGTCCGGTGAG	405 bp
		R: TTAGCCACAGAGTACTTTGCTGTCA	
*β-actin*	NM_031144	F: TCATGAAGTGTGACGTTGACATCCGT	285 bp
		R: CCTAGAAGCATTTGCGGTGCACGATG	

Primers were used for the determination of mRNA expression levels in TR-iBRB2 cells by quantitative real-time PCR analysis.

### Data analysis

Unless otherwise indicated, all data represent mean±SEM. An unpaired, two-tailed Student's *t*-test was used to determine the significance of differences between two groups. Statistical significance of differences among means of several groups was determined by one-way ANOVA followed by the modified Fisher's least-squares difference method.

## Results

**Figure 2:**
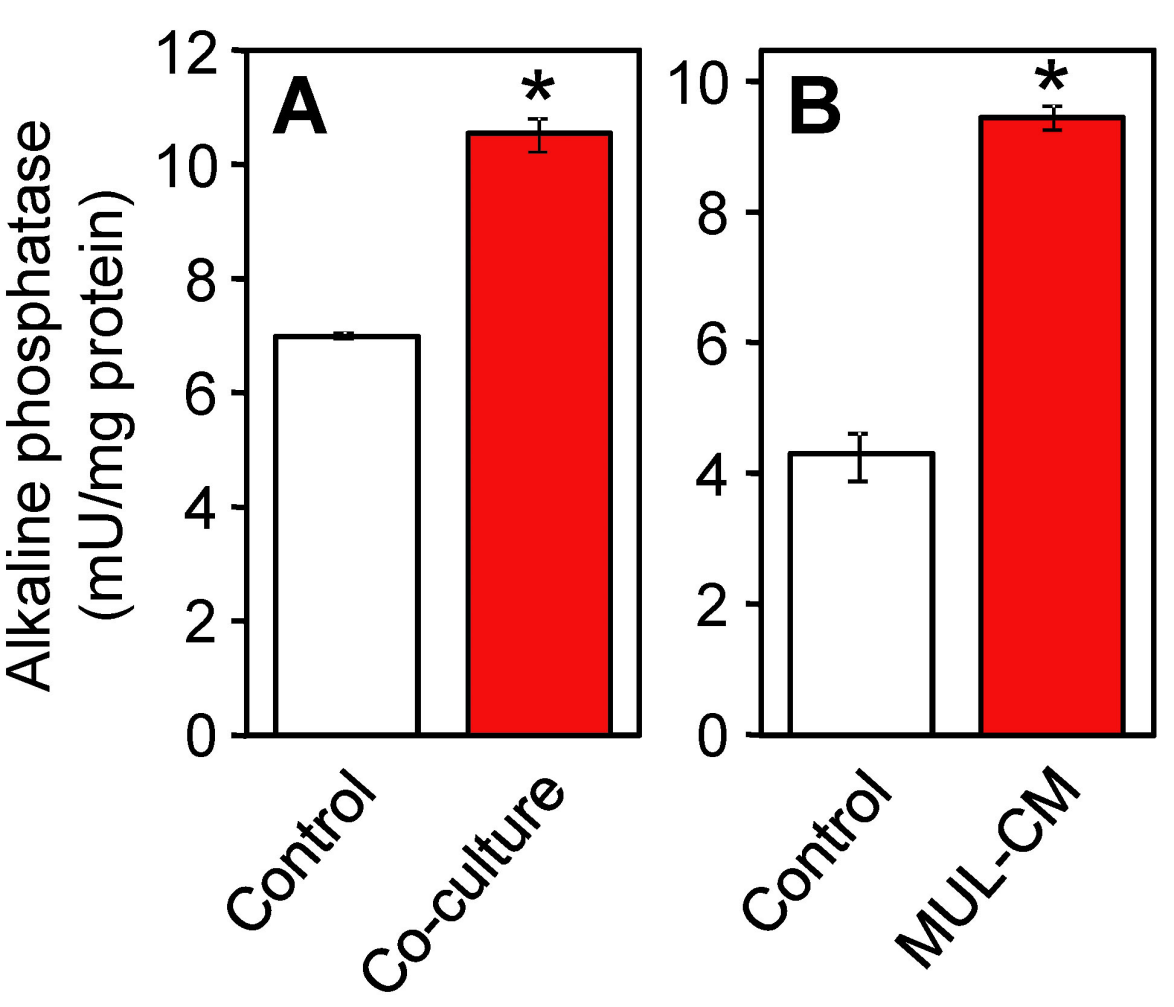
Alkaline phosphatase activity in TR-iBRB2 cells. Effects of transfilter co-culture with TR-MUL5 cells (**A**) and soluble factors secreted from TR-MUL5 cells (**B**) on the activity of alkaline phosphatase in TR-iBRB2 cells. **A:** TR-MUL5 cells were situated on the basolateral aspect of TR-iBRB2 cell monolayers as might occur in vivo and cells were co-cultured for six days. **B:** TR-iBRB2 cells were cultured in the conditioned medium of TR-MUL5 cells (MUL-CM) for 24 h. Each column represents the mean±SEM (n=3). Asterisk represents p<0.01, significantly different from the control.

Alkaline phosphatase in endothelial cells is induced by glial cells [17,18]. To determine whether interactions occur between TR-iBRB2 cells and TR-MUL5 cells, we examined the effects of transfilter co-culture with TR-MUL5 cells and soluble factors secreted from TR-MUL5 cells on the activity of alkaline phosphatase in TR-iBRB2 cells. In the transfilter co-culture, TR-MUL5 cells were situated on the basolateral aspect of the TR-iBRB2 cell monolayer as might occur in vivo (Figure 1A) and cells were cocultured for six days. The effect of soluble factors secreted from TR-MUL5 cells were examined by culturing TR-iBRB2 cells in fivefold concentrated conditioned medium of TR-MUL5 cells (MUL-CM) for 24 h (Figure 1B). As shown in Figure 2, alkaline phosphatase activity in TR-iBRB2 cells was significantly increased to 153% by coculture with TR-MUL5 cells and 222% by coculture with MUL-CM cells.

To identify genes, the expressions of which are modulated by soluble factors secreted from TR-MUL5 cells, we incubated TR-iBRB2 cells with MUL-CM for 24 h and subjected them to microarray analysis for genome-wide gene expression profiling. We compared the gene expression levels between control and MUL-CM treatment groups and identified genes showing differential expression with at least a threefold change. As shown in Table 2, MUL-CM treatment induced the expression of five genes, namely, myosin heavy chain polypeptide 6 (*Myh6*), retinol binding protein 1 (*Rbp1*), metallothionein 1a (*Mt1a*), ectonucleotide pyrophosphatase/phosphodiesterase 1 (*Enpp1*), and plasminogen activator inhibitor 1 (*PAI-1*), and suppressed the expression of an inhibitor of DNA binding 2 (*Id2*) gene.

**Table 2:** Genes modulated by TR-MUL5 conditioned medium in TR-iBRB2 cells

**Gene**	**Accession number**	**Fold change**
Myosin heavy chain polypeptide 6 (*Myh6*)	NM_017239	16
Retinol binding protein 1 (*Rbp1*)	NM_012733	5.3
Metallothionein 1a (*Mt1a*)	NM_138826	5.3
Ectonucleotide pyrophosphatase/phosphodiesterase 1 (*Enpp1*)	NM_053535	4.9
Plasminogen activator inhibitor 1 (*PAI-1*)	NM_012620	3.5
Inhibitor of DNA binding 2 (*Id2*)	NM_013060	0.23

TR-iBRB2 cells were incubated with MUL-CM for 24 h and subjected them to microarray analysis. We compared the gene expression levels between control and MUL-CM treatment groups and identified genes showing differential expression with at least a threefold change.

**Figure 3:**
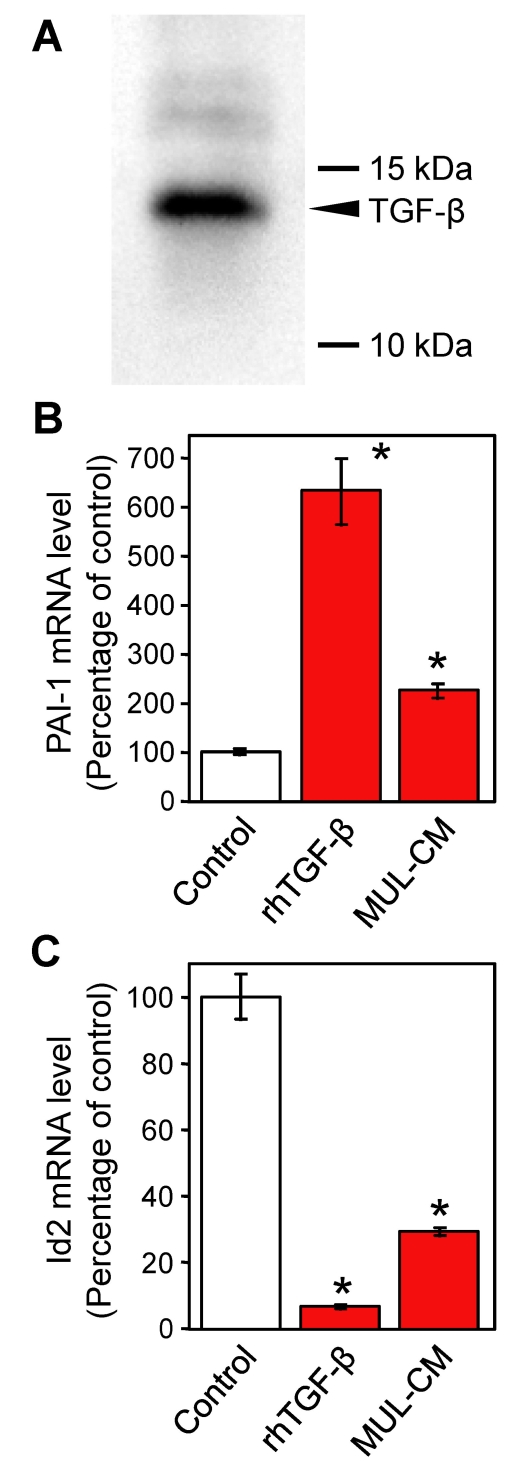
TGF-β, *PAI-1*, and *Id2* expressions. Expression of TGF-β1 in the conditioned medium of TR-MUL5 cells (MUL-CM) (**A**) and modulation of *PAI-1* (**B**) and *Id2* (**C**) mRNA expressions by recombinant human TGF-β1 (rhTGF-β1) and MUL-CM in TR-iBRB2 cells. **A:** The expression of TGF-β1 was determined by immunoblot analysis. **B, C:** The expression levels of *PAI-1* and *Id2* mRNA were determined by quantitative real-time PCR analysis and normalized to *β-actin* mRNA expression. Each column represents the mean±SEM (n=4–12). Asterisk represents p<0.01, significantly different from the control.

*PAI-1* and *Id2* are anti-angiogenic and pro-angiogenic factors, respectively, but both proteins are regulated by transforming growth factor β (TGF-β) signaling pathways in endothelial cells [19-21]. The expression of TGF-β1 protein in MUL-CM was examined by immunoblot analysis. As shown in Figure 3A, a band around 12.5 kDa was detected in MUL-CM, indicating that TR-MUL5 cells secret TGF-β1 protein. Quantitative real-time PCR analysis was performed to confirm the effect of TGF-β1 on the expression of *PAI-1* and *Id2* mRNAs in TR-iBRB2 cells (Figures 3B,C). Treatment with 2 ng/ml recombinant human TGF-β1 for 24 h resulted in an increase in *PAI-1* mRNA of 520% and a decrease in *Id2* mRNA of 93.2%. These data are consistent with TR-iBRB2 cells incubated with MUL-CM for 24 h (122% increase in *PAI-1* and 70.8% decrease in *Id2*).

## Discussion

The present study demonstrated that TR-MUL5 cell-derived factors modulate alkaline phosphatase activity and the expression of several genes including *PAI-1* and *Id2* in TR-iBRB2 cells. Endothelial cells that are present in the gliovascular unit (e.g., blood-brain barrier [BBB] and inner BRB) are known to be especially abundant in alkaline phosphatase [22]. The observed induction of alkaline phosphatase in TR-iBRB2 cells by TR-MUL5 cell-derived factor (Figure 2) suggested that our cell culture model of the inner BRB is appropriate for the analysis of the paracrine interaction between Müller and retinal capillary endothelial cells. Moreover, both co-culture with TR-MUL5 cells and MUL-CM induced alkaline phosphatase activity in TR-iBRB2 cells (Figure 2), implying that the diffusive signal is predominantly involved in the induction of alkaline phosphatase at the inner BRB. This is consistent with studies using in vitro cell culture models of the BBB, in which a diffusive signal by glia-derived factors, including basic fibroblast growth factor, is suggested to induce endothelial alkaline phosphatase [17,18]. Following treatment with MUL-CM, TR-iBRB2 cells increased the expression of *Enpp1* (Table 2). *Enpp1* and alkaline phosphatase overlap in phosphatase function as well as act together in the extracellular hydrolysis of ATP to inorganic phosphate [23,24]. Therefore, *Enpp1* might contribute the induction of alkaline phosphatase by MUL-CM via its phosphatase activity.

Microarray analysis demonstrated that *PAI-1* and *Id2* in TR-iBRB2 cells are respectively induced and suppressed by MUL-CM (Table 2), which is further confirmed by quantitative real-time PCR analysis (Figure 3). We also demonstrated that *PAI-1* and *Id2* in TR-iBRB2 cells are respectively induced and suppressed by TGF-β1 (Figures 3B,C), which is seen to be secreted from TR-MUL5 cells (Figure 3A). In agreement with our results, it has already shown that TGF-β is secreted from rat [25] and human [26] Müller cells. These results raise the possibility that Müller cells may modulate retinal angiogenesis by altering its secretion of TGF-β1, although further studies are needed to confirm the involvement of TGF-β1 as a paracrine factor between Müller and endothelial cells. It is also necessary to determine the effect of MUL-CM and TGF-β1 on cell migration and proliferation in TR-iBRB2 cells.

*Myh6* encodes the cardiac α-myosin heavy chain and its expression is reported to be induced by TGF-β signaling pathways in cardiomyocytes during embryonic heart development [27], but the physiologic significance of *Myh6* in retinal endothelial cells still needs to be clarified. *Rbp1* in cultured hepatic stellate and fibroblast cells [28,29] and *Mt1a* in retinal pigment epithelial cells [30] are induced by TGF-β. *Rbp1* binds to retinol in the cytoplasm and is involved in the intracellular transport of retinol in a variety of cells including retinal pigment epithelial cells. *Mt1a* protects cells against oxidative stress and apoptosis in the retina. *Enpp1* is induced by TGF-β and bFGF in osteosarcoma cells [31,32]. Therefore, paracrine interactions between Müller and endothelial cells seem to play an important role in modulating several functions, such as intracellular retinol transport and protection against oxidative stress, in retinal capillary endothelial cells.

In conclusion, Müller cells appear to act as one of the modulators of retinal angiogenesis and TGF-β may be involved in this modulation. To our knowledge, this is the first report to suggest that Müller cells modulate the expression of *PAI-1* and *Id2* in retinal endothelial cells. As the number of diabetic patients is increasing throughout the world, acquired blindness in diabetic retinopathy is a serious problem. The current findings offer important information for a better understanding of the mechanism of retinal neovascularization which is a common pathological finding in diabetic retinopathy.
